# Complex interactions of gut-derived short-chain fatty acids in hyperuricemia and gout pathophysiology

**DOI:** 10.3389/fmicb.2026.1772631

**Published:** 2026-03-05

**Authors:** Yujiang Cui, Wei Sun, Lijuan Wei, Shuang Fan, Qian Li, Liwei Duan

**Affiliations:** Department of Gastroenterology and Digestive Endoscopy Center, The Second Hospital of Jilin University, Changchun, China

**Keywords:** gut microbiota, hyperuricemia, prebiotics, probiotics, short-chain fatty acids, uric acid transporters

## Abstract

Hyperuricemia is a common metabolic disorder associated with gout, kidney injury, cardiovascular disease, and chronic low-grade inflammation. Increasing evidence indicates that abnormalities in intestinal uric acid handling and gut microbial metabolism contribute substantially to systemic urate imbalance, particularly when renal excretion is impaired. Among microbiota-derived metabolites, short-chain fatty acids (SCFAs) have emerged as key regulators linking gut microbial ecology with uric acid metabolism through coordinated effects on epithelial barrier integrity, inflammatory signaling, and urate transport. Growing interest in prebiotics and probiotics has further highlighted the therapeutic potential of targeting SCFAs production as a complementary strategy to traditional urate-lowering drugs. Given that hyperuricemia is the primary pathogenic precursor to gout, this review also examines the role of SCFAs in modulating gout-associated inflammation. This review integrates current findings on the microbiota-SCFA-urate axis and outlines how SCFA-centered gut modulation may provide a viable framework for managing hyperuricemia and gout.

## 1 Introduction

Hyperuricemia (HUA) is an increasingly prevalent metabolic disorder worldwide, with its incidence rising in parallel with changes in dietary habits and lifestyle (Du et al., 2024; Feng et al., 2024). HUA is defined as a serum uric acid (SUA) concentration exceeding 7.0 mg/dl and constitutes a major risk factor not only for gout but also for chronic kidney disease, hypertension, cardiovascular disease, and metabolic syndrome (Dalbeth et al., 2021; Johnson et al., 2018; So and Thorens, 2010). Uric acid homeostasis depends on a balance between its production and excretion. Uric acid is primarily generated through purine metabolism catalyzed by xanthine oxidase (XOD), with approximately 70% excreted via the kidneys and 30% eliminated through the intestine (Maiuolo et al., 2016; Sorensen, 1965). Conventional explanations of HUA emphasize excessive uric acid production or reduced renal excretion, and current strategies primarily target XOD inhibition or enhancement of renal urate excretion (Boss and Seegmiller, 1979).

Recent research has revealed that the intestine plays a more substantial role in uric acid homeostasis than previously recognized, particularly under conditions of renal impairment, where intestinal excretion becomes a compensatory mechanism (Yin et al., 2022; Singh et al., 2024). A growing body of studies links hyperuricemia to significant gut microbiota dysbiosis, including reductions in microbial diversity, depletion of beneficial commensals, and proliferation of opportunistic pathogens (Li et al., 2016; Liu et al., 2025; Guo et al., 2016; Chu et al., 2021). Among gut microbial metabolites, short-chain fatty acids (SCFAs) such as acetate, propionate, and butyrate have attracted considerable attention for their capacity to influence both uric acid metabolism and inflammatory responses (Koh et al., 2016). SCFAs are generated through microbial fermentation of dietary fibers and contribute to maintaining epithelial barrier function, immune balance, and urate regulatory pathways (Koh et al., 2016; Sun et al., 2024; Dalile et al., 2019). Notably, dietary patterns commonly observed in individuals with HUA, including increased intake of purine-rich foods and reduced fiber consumption, are associated with diminished SCFAs production and may exacerbate uric acid dysregulation (Sun et al., 2024).

**Figure 1:**
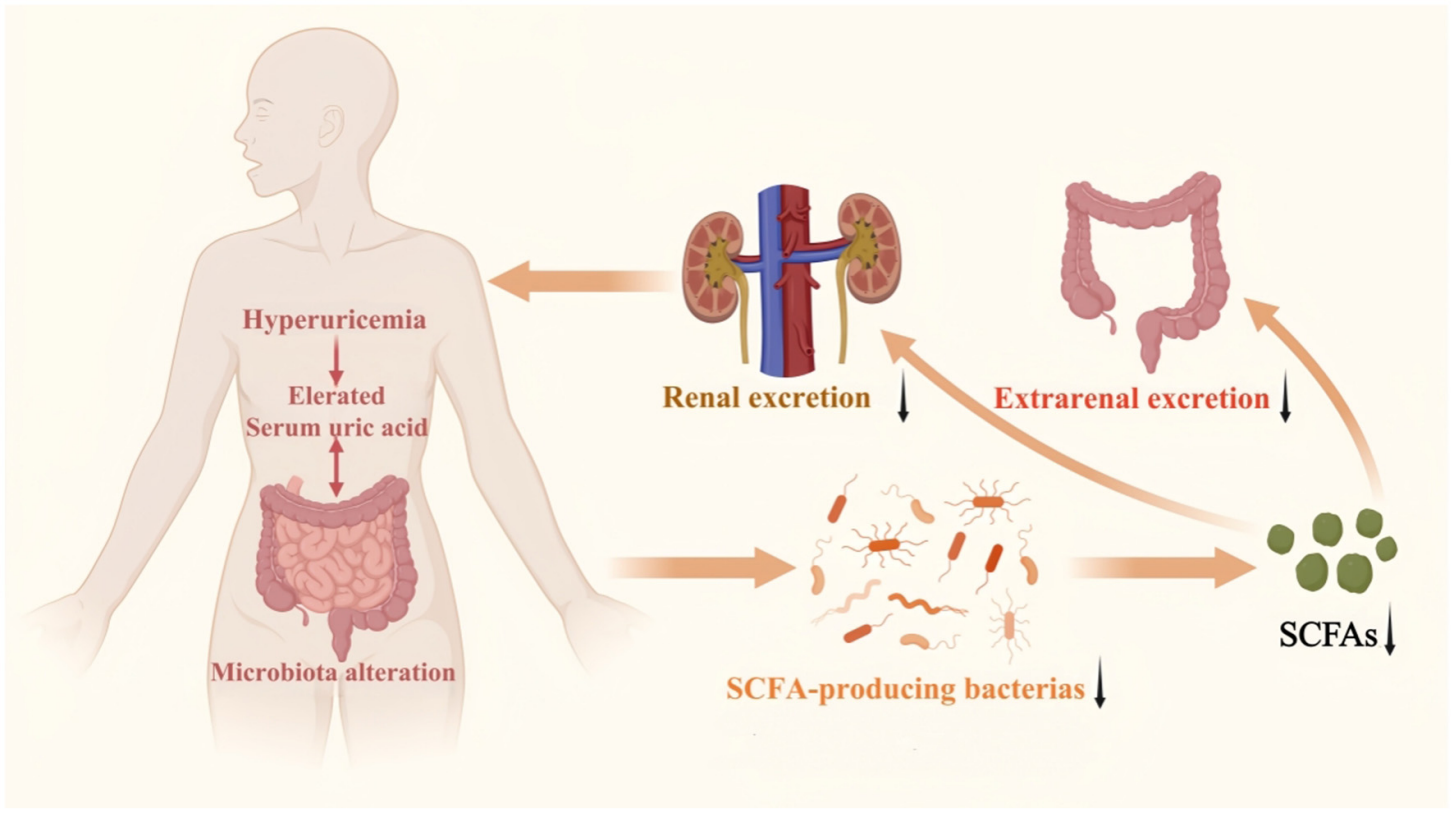
Schematic diagram of the role of gut microbiota and its metabolite SCFAs in hyperuricemia. Elevated serum uric acid levels lead to alteration in the gut microbiota, resulting in reduced production of SCFAs. This decrease in SCFAs ultimately diminishes both renal and extrarenal (primarily intestinal) excretion of uric acid, thereby further exacerbating hyperuricemia.

Given these insights, understanding the mechanisms by which gut microbiota and SCFAs influence uric acid production, transport, excretion, and inflammatory activation has become essential. Importantly, hyperuricemia serves as the critical precursor to gout. Therefore, elucidating how SCFAs modulate not only uric acid levels but also gout-related inflammatory pathways is of clinical relevance. This review summarizes current advances in gut microbial alterations, SCFA-mediated regulation of urate transporters and xanthine oxidase, modulation of intestinal barrier function, and inflammatory pathways relevant to hyperuricemia and gout. It further discusses emerging microbiota-targeted strategies, including prebiotics and probiotics, that harness SCFA-mediated effects for the prevention and treatment of hyperuricemia and its progression to gout. The proposed interplay between gut microbiota, SCFAs, and uric acid excretion is summarized in Figure 1.

## 2 Hyperuricemia and gout

### 2.1 Epidemiology

Hyperuricemia is a metabolic disorder primarily arising from disturbances in purine metabolism, characterized by persistently elevated levels of uric acid in the body (Dalbeth et al., 2021). As the terminal product of human purine catabolism, uric acid is normally maintained in a dynamic balance between endogenous synthesis and systemic excretion (Maiuolo et al., 2016). The increasing global prevalence of hyperuricemia is largely attributed to excessive dietary intake of purine-rich foods, particularly red meat, seafood, fructose, and alcohol, which collectively enhance the body's uric acid burden (Choi, 2010). This delicate homeostasis can be disrupted under various pathological conditions, such as accelerated hepatic metabolism, increased cellular turnover, or impaired renal and intestinal excretion, ultimately leading to hyperuricemia (Dalbeth et al., 2018).

A growing body of epidemiological and clinical evidence indicates that hyperuricemia is closely associated with a spectrum of metabolic and cardiovascular disorders, including acute and chronic kidney disease, hypertension, cardiovascular disease, type 2 diabetes mellitus, and dyslipidemia (Johnson et al., 2018; Ponticelli et al., 2020; Feig et al., 2008; Kim et al., 2015; El Ridi and Tallima, 2017). The stage characterized by elevated serum urate levels in the absence of clinical symptoms is defined as asymptomatic hyperuricemia. Although most individuals with hyperuricemia remain asymptomatic, approximately 10% eventually develop symptomatic gout (Dalbeth et al., 2015).

### 2.2 Pathophysiology

Gout is an inflammatory arthritis that directly arises from hyperuricemia and is pathologically characterized by the deposition of monosodium urate (MSU) crystals within joints and periarticular tissues (Dalbeth et al., 2016). These crystals are potent activators of the NLRP3 inflammasome, triggering a robust inflammatory cascade that culminates in acute gout flares (Martinon et al., 2006). The NLRP3 inflammasome is a multiprotein complex composed of NLRP3, the adaptor protein ASC, and caspase-1, whose principal function is to mediate the proteolytic activation of interleukin-1β (IL-1β) (Broz and Dixit, 2016). Inflammasome activation follows a canonical two-signal model: the priming signal involves Toll-like receptor (TLR4 and TLR2) activation, while the subsequent activation signal can be elicited by ionic flux, reactive oxygen species (ROS) generation, or lysosomal destabilization (He et al., 2016; Bauernfeind et al., 2009). Both MSU crystals and soluble urate (sUA) have been shown to activate the NLRP3 inflammasome through partially distinct pathways (Kelley et al., 2019; Schorn et al., 2011; Martinon, 2010; Braga et al., 2017; Kimura et al., 2020), ultimately leading to IL-1β maturation and the recruitment of inflammatory cells (Martinon et al., 2002). Beyond canonical NLRP3 inflammasome activation, gouty inflammation is orchestrated by a complex network of interconnected innate immune pathways. Recent studies have also demonstrated an essential role for complement activation in gout pathogenesis. MSU crystals directly trigger the classical and alternative complement pathways, leading to the generation of C3a and C5a anaphylatoxins, which enhance vascular permeability and act as potent chemoattractants for neutrophils and monocytes (Wessig et al., 2022). In addition to IL-1β, a broad cytokine and chemokine network contributes to gout flares. MSU-stimulated macrophages and neutrophils produce tumor necrosis factor-α, interleukin-6, interleukin-8, and granulocyte colony-stimulating factor, which collectively sustain leukocyte recruitment, endothelial activation, and synovial inflammation (Chen et al., 2006; Busso and So, 2010).

Interestingly, acute gout attacks are typically self-limiting and resolve through several endogenous mechanisms. These include the formation of neutrophil extracellular traps (NETs) by aggregated neutrophils, normalization of serum urate levels, clearance of inflammatory cells, upregulation of transforming growth factor-β1 (TGF-β1), modulation of the pro- and anti-inflammatory cytokine environment, and the participation of specialized pro-resolving lipid mediators (Schauer et al., 2014; Steiger and Harper, 2014). As hyperuricemia remains a principal trigger for gout flares, elucidating these resolution pathways provides crucial insights for the development of targeted curative interventions.

## 3 Microbial contributions to uric acid metabolism

The gut microbiota orchestrates systemic uric acid homeostasis through multifaceted mechanisms, and its disruption is an established pathogenic factor in hyperuricemia development and progression (Li et al., 2016). Compelling evidence from fecal microbiota transplantation (FMT) studies demonstrates that transferring gut microbiota from hyperuricemic donors to normouricemic recipients significantly elevates serum uric acid levels in the latter, providing direct causal evidence for the indispensable involvement of intestinal microbes in hyperuricemia pathogenesis (Liu et al., 2020). These findings position the gut microbiota not as a passive bystander but as an active modulator of purine metabolism and uric acid balance.

### 3.1 Gut microbiota dysbiosis in hyperuricemia and gout

Characteristic and reproducible alterations in gut microbial composition, commonly termed dysbiosis, have been consistently observed in both human hyperuricemic cohorts and experimental models. Affected individuals typically exhibit reduced microbial α-diversity, diminished abundance of anti-inflammatory commensal taxa, and enrichment of opportunistic pathogens and pro-inflammatory genera (Wei et al., 2022; Pan et al., 2020). Comparative metagenomic analyses of gout patients reveal an elevated Bacteroidetes-to-Firmicutes ratio, accompanied by an overrepresentation of inflammatory genera such as *Prevotella, Fusobacterium*, and *Bacteroides*. In contrast, there is a marked decline in bacterial species capable of direct uric acid degradation (e.g., via the uricase pathway) and in SCFA-producing bacteria such as *Roseburia* and *Faecalibacterium*. This compositional shift generates a metabolic and immunological milieu that favors systemic uric acid accumulation and sustains inflammation (Chu et al., 2021).

A critical functional distinction exists between the gut microbiota of individuals with asymptomatic hyperuricemia (asHU) and those with symptomatic gout. Individuals with ASHU exhibit significantly higher abundances of SCFA-producing bacteria, which are known for generating butyrate and propionate (Martínez-Nava et al., 2023). These metabolites exert potent anti-inflammatory and immunomodulatory effects, potentially conferring protection against progression from asymptomatic hyperuricemia to overt gout. Supporting this notion, microbial diversity and SCFA-producer abundance are consistently reduced in gout patients compared with asHU individuals (Kim et al., 2022). Moreover, pharmacological interventions such as the xanthine oxidase inhibitor febuxostat have been shown to partially restore gut microbial diversity and beneficial taxa in hyperuricemic models, underscoring the bidirectional interplay between host purine metabolism, serum uric acid levels, and intestinal microbial ecology (Lin et al., 2021).

Mechanistically, the gut microbiota contributes directly to host uric acid metabolism through multiple, complementary enzymatic pathways. Certain bacterial strains, such as *Lactobacillus plantarum* SQ001 isolated from the cecum, facilitate the uptake and hydrolysis of purine nucleotides via intrinsic nucleoside hydrolase activity, thereby reducing the substrate pool for *de novo* uric acid synthesis (Fu et al., 2024). Other *Lactobacillus* species employ distinct purine-degrading enzymes to decrease uric acid production while enhancing microbial degradation (Yamada et al., 2016; Li et al., 2014). Furthermore, numerous intestinal bacteria harbor conserved gene clusters enabling the anaerobic metabolism of uric acid into downstream products such as xanthine and SCFAs, constituting a key microbial pathway for intestinal uric acid disposal and energy recovery (Liu Y. et al., 2023). The physiological gradient of uric acid concentrations along the intestinal tract, higher in the relatively sterile upper intestine and lower in the microbially dense distal colon, further highlights the active and substantial contribution of the gut microbiota to local uric acid catabolism and systemic homeostasis (Yun et al., 2017).

### 3.2 Microbial metabolites in the regulation of uric acid homeostasis

The gut microbiota profoundly influences systemic uric acid homeostasis through the production of a wide array of metabolites that directly and indirectly modulate purine metabolism, urate transport, and inflammatory responses. Comprehensive metabolomic profiling of serum and fecal samples from gout patients reveals significant and characteristic alterations, typically characterized by upregulated levels of metabolites such as glycine, taurine, succinate, acetate, α-glucose, and β-glucose, alongside the downregulation of valine, asparagine, and aspartate (Shao et al., 2017). These patients also exhibit signatures of enhanced carbohydrate metabolism, with elevated concentrations of glucose, TCA cycle intermediates, pyruvate, and lactate, indicating a profound reprogramming of host and/or microbial energy metabolism that is intricately linked with the hyperuricemic state (Ohashi et al., 2024).

Key microbial and host-derived metabolites serve dual, interconnected roles in both cellular energy provision and uric acid regulation. Acetate, succinate, and glucose, for instance, contribute to cellular energy metabolism via ATP generation, while this ATP may simultaneously facilitate active uric acid excretion processes in intestinal epithelial cells (Bauernfeind et al., 2009). Conversely, glycine and aspartate are essential substrates for purine synthesis; thus, their altered levels may disrupt purine metabolism and promote endogenous uric acid production (Méndez-Salazar and Martínez-Nava, 2022). Furthermore, specific metabolites correlate with clinical indicators: glycine and tryptophan show a positive correlation with serum uric acid levels (Zhu et al., 2023), while taurine and succinate participate in inflammatory responses through distinct mechanisms (Tannahill et al., 2013; Patel et al., 2016). In addition to host metabolites, gut microbiota-derived components actively drive pathogenic processes. Lipopolysaccharide (LPS), a major component of the outer membrane of Gram-negative bacteria, emerges as a crucial mediator in hyperuricemia progression and its inflammatory complications. Elevated systemic LPS, often resulting from increased intestinal permeability, enhances the activity of hepatic xanthine oxidase, a key enzyme, thereby promoting endogenous uric acid production (Deitch et al., 1989; Hassoun et al., 1998). Furthermore, LPS may additionally increase gout risk by inducing low-grade inflammation and potentially impairing renal function and urate excretion (Xi et al., 2019). Through its lipid A component, which acts as a classic pathogen-associated molecular pattern (PAMP), LPS activates Toll-like receptor 4 (TLR4) signaling on immune and other cells, triggering the release of pro-inflammatory cytokines such as IL-1β and TNF-α, thereby establishing a direct mechanistic link between gut barrier integrity, innate immune activation, and systemic inflammation in gout (Lu et al., 2008). In contrast, certain microbial metabolites offer protective benefits. SCFAs, particularly butyrate, propionate, and acetate, demonstrate significant beneficial effects on uric acid metabolism and associated inflammation. The reduction in colonic luminal uric acid following butyrate administration implicates this SCFAs directly in intestinal uric acid handling, whether through modulation of microbial metabolism or host transporter expression (Hamer et al., 2009). In murine models of gouty arthritis, interventions such as high-fiber diets (which boost endogenous SCFAs production) and direct acetate supplementation effectively attenuate monosodium urate crystal-induced neutrophilic inflammation, suggesting that SCFAs can modulate the inflammatory processes central to acute gout attacks (Vieira et al., 2017).

These collective findings establish the gut microbiome as a key modulator of gout pathogenesis through its metabolites, which act on multiple fronts including regulating purine metabolism, influencing uric acid dynamics, and shaping inflammatory responses. Exploiting these mechanisms, particularly the beneficial effects of SCFAs, offers a promising therapeutic avenue for hyperuricemia and gout.

### 3.3 Intestinal uric acid excretion: the role of transporters

Intestinal uric acid excretion is mediated by a complex network of specific transporter systems expressed on the apical and basolateral membranes of enterocytes, which work in concert to regulate systemic uric acid levels. While renal urate transporters have been extensively characterized over decades, their intestinal counterparts represent an emerging and highly dynamic research focus with substantial potential for managing hyperuricemia, offering a substitute excretory route (So and Thorens, 2010; Wright et al., 2010).

ATP-binding cassette sub-family G member 2 (ABCG2), also known as the breast cancer resistance protein, functions as a high-capacity urate efflux transporter located primarily on the apical membrane of intestinal epithelial cells (Dean et al., 2001; Matsuo et al., 2009). It is highly expressed in the duodenum, jejunum, and colon (Maliepaard et al., 2001), and demonstrates dynamic regulation in response to physiological and pathological challenges. During acute gout attacks in mouse models, intestinal ABCG2 protein and mRNA expression are significantly upregulated, resulting in a compensatory enhancement of intestinal uric acid excretion, suggesting a physiological adaptive response to uric acid overload. Conversely, mice lacking the ABCG2 gene show a marked decline in intestinal uric acid excretion, confirming the transporter's indispensable and non-redundant function in this extra-renal pathway (Zhao et al., 2023). Complementary pharmacological evidence further confirms this function; for instance, inhibition of intestinal BCRP/ABCG2 with specific inhibitors like elacridar substantially decreases endogenous uric acid secretion into the gut lumen, while regional variations in uric acid excretion rates along the intestine closely parallel the gradient of BCRP mRNA expression levels (Hosomi et al., 2012). Studies using oxonate-treated mouse models, which inhibit uricase, further corroborate these findings by demonstrating that reduced intestinal urate excretion directly contributes to serum uric acid elevation, highlighting the physiological relevance of this pathway (Ichida et al., 2012).

Solute Carrier Family 2 Member 9 (SLC2A9), which encodes the facilitated glucose transporter GLUT9, is a key determinant of intestinal urate handling. Although its critical function in renal tubular urate reabsorption is well-established (Caulfield et al., 2008; Vitart et al., 2008), GLUT9 is also abundantly expressed in intestinal tissues, particularly in the jejunum and ileum (DeBosch et al., 2014). Evidence from genetically engineered mouse models indicates that mice with intestinal epithelial cell-specific GLUT9 deficiency show significantly reduced intestinal urate clearance rates, suggesting an important contribution of intestinal GLUT9 to overall uric acid excretion, although the precise subcellular localization and mechanistic details of its action in the gut require further validation (DeBosch et al., 2014).

Beyond these two relatively well-characterized transporters, emerging evidence suggests that additional transport systems participate in the complex process of intestinal urate handling. These include Monocarboxylate transporter 9 (MCT9), sodium/phosphate cotransporters (SLC34 family), and organic anion transporter 10 (OAT10), all of which have been implicated in uric acid transport in various tissues and may contribute to intestinal uric acid excretion or absorption Z. However, their specific roles, relative quantitative contributions, and regulatory mechanisms within the intestinal epithelium remain to be fully elucidated.

The collective evidence firmly establishes intestinal urate transporters, particularly ABCG2, as crucial physiological determinants of uric acid excretion and, consequently, serum uric acid regulation. While ABCG2 and GLUT9 have emerged as key players, a comprehensive and integrated understanding of transporter interactions, their transcriptional and post-translational regulatory mechanisms, and their adaptations to various physiological, dietary, and pathological conditions represents an important frontier for future research. These investigations hold particular promise for developing novel therapeutic strategies that target the enhancement of intestinal urate excretion for the management of hyperuricemia and gout, potentially offering benefits for patients with renal urate excretion impairment.

## 4 SCFAs: key mediators of uric acid balance

### 4.1 SCFAs production and interactions with the gut microbiota

SCFAs are the primary metabolites produced by the gut microbiota through the anaerobic fermentation of dietary fibers, predominantly within the cecum and colon. They play crucial and diverse roles in host physiology, including regulating the immune system, maintaining intestinal homeostasis, restoring the integrity of the intestinal barrier, facilitating intracellular signal transduction, and broadly influencing host metabolism. As key effector molecules of the gut microbiota, these metabolites are intimately linked to human health and disease states (Koh et al., 2016). The most abundant SCFAs are acetate, propionate, and butyrate, each with distinct biological functions (Topping and Clifton, 2001). The production of SCFAs is primarily dependent on the microbial fermentation of non-digestible polysaccharides (NDPs) derived from plant cell walls, encompassing both soluble and insoluble dietary fibers. However, patients with hyperuricemia often exhibit dietary modifications, such as reduced consumption of vegetables and increased intake of purine-rich meats (Sun et al., 2024), which can lead to a diminished substrate availability for microbial fermentation, thereby indirectly reducing the intestinal production of SCFAs. Epidemiological and experimental studies consistently show that the abundance of SCFA-producing bacteria, such as *Bifidobacterium, Prevotella*, and *Ruminococcus*, is negatively correlated with serum uric acid levels, pointing to their protective properties (Guo et al., 2016; Xie X. Q. et al., 2021).

Beyond their direct metabolic effects, SCFAs contribute to maintaining a balanced gut microbiota ecosystem by inhibiting the colonization and growth of pathogenic bacteria, such as *Salmonella* and *Escherichia coli*, while simultaneously promoting the proliferation of beneficial bacteria like *Roseburia, Blautia*, and *Ruminococcus* (Fusco et al., 2023; Baltazar-Díaz et al., 2022). The administration of specific dietary polysaccharides can be strategically used to boost SCFA levels. For instance, inulin, a fermentable fiber, is efficiently metabolized by gut bacteria to yield acetate, propionate, and butyrate (Tawfick et al., 2022). Intervention studies have demonstrated that inulin treatment in hyperuricemic mice significantly increased fecal concentrations of these SCFAs, improved overall gut microbiota diversity, and enriched the abundance of probiotic genera, including *Ruminococcus* and *Bifidobacterium* (Guo et al., 2021). Similarly, konjac glucomannan (KGM) is fermented into SCFAs, including acetate, propionate, butyrate, and valerate. Following KGM administration, hyperuricemic patients exhibited a significant rise in total SCFA levels, particularly butyrate and valerate, accompanied by a reversal of gut microbiota dysbiosis. This was characterized by an increase in beneficial bacteria such as *Faecalibacterium, Ruminococcus*, and *Lachnoclostridium*, and a reduction in the abundance of certain pathogenic taxa (Deng et al., 2024).

SCFA-producing bacteria and their metabolites are beneficial in mitigating hyperuricemia and gout. They significantly lower serum uric acid levels, help restore a healthy gut microbiota composition, and may inhibit the progression from asymptomatic hyperuricemia to clinical gout. SCFAs are thus crucial regulators of gut microbiota balance and intestinal health in the context of hyperuricemia. Nevertheless, research directly elucidating the causal effects of specific SCFAs on the gut microbial community in hyperuricemic patients remains limited, warranting further exploration to fully understand their potential and mechanisms of action.

### 4.2 SCFAs and urate transporters

Systemic uric acid levels are critically determined by the balance between renal reabsorption and excretion, processes mediated by specific urate transporters. Key renal reabsorption transporters include urate anion transporter 1 (URAT1) and glucose transporter 9 (GLUT9), while excretion involves transporters such as organic anion transporter 1 (OAT1), OAT3, and the ATP-binding cassette transporter ABCG2 (So and Thorens, 2010). A growing body of evidence indicates that SCFAs can influence uric acid homeostasis by modulating the function of these transporters. Molecular docking studies suggest that acetate, propionate, and butyrate can physically bind to URAT1 and GLUT9. Supporting this, *in vitro* functional assays have demonstrated that SCFAs dose-dependently inhibit urate transport by both URAT1 and GLUT9, thereby providing mechanistic evidence for reduced renal uric acid reabsorption (Li Y. et al., 2022). Furthermore, sodium-coupled monocarboxylate transporters (SMCTs), which transport monocarboxylates like lactate, can enhance URAT1-mediated urate reabsorption by providing an exchangeable anion. Notably, butyrate and propionate act as inhibitors of SMCTs, thereby weakening this synergistic reabsorptive mechanism and further reducing uric acid reabsorption via URAT1 (Lu et al., 2013). Given that URAT1 and GLUT9 are responsible for the majority of urate reabsorbed into the bloodstream, SCFAs ultimately promote urinary uric acid excretion by inhibiting these proteins.

SCFAs also impact intestinal urate excretion. Butyrate, in particular, serves as a preferred energy source for colonic epithelial cells, potentially supporting the energy-demanding process of active urate secretion (Dalile et al., 2019). In hyperuricemic mouse models, a reduction in intestinal ABCG2 expression is commonly observed. However, treatment with sodium butyrate was shown to significantly upregulate intestinal ABCG2 protein expression, enhance intestinal uric acid excretion, and consequently lower serum uric acid levels (Li et al., 2023). The mechanistic underpinnings of this upregulation involve several pathways. SCFAs are known to stimulate the activation of the nuclear receptor peroxisome proliferator-activated receptor gamma (PPARγ) (Alex et al., 2013), and butyrate has been specifically shown to induce the expression of BCRP/ABCG2 in the rat intestine via the PPARγ signaling pathway (Xie Q. S. et al., 2021). Additionally, sodium butyrate activates AMP-activated protein kinase (AMPK) (Li X. et al., 2022), which can upregulate ABCG2 expression through the AMPK/AKT/cAMP response element-binding protein (CREB) axis (Xue et al., 2024). This activation enhances the binding of CREB to the ABCG2 promoter, leading to increased transcription of the transporter (Lu et al., 2020).

In summary, SCFAs regulate urate transporters in both the kidneys and the intestines. They reduce renal uric acid reabsorption by inhibiting URAT1 and GLUT9 and increase uric acid excretion by upregulating intestinal ABCG2. This multi-faceted action effectively lowers serum uric acid levels, positioning SCFAs as critical endogenous mediators and promising agents for improving hyperuricemia. The distinct roles and mechanistic profiles of major SCFAs in regulating uric acid homeostasis and inflammation are systematically compared in Table 1.

**Table 1:** Mechanisms of major SCFAs in regulating uric acid homeostasis and gout-associated inflammation.

**SCFA**	**Key mechanisms in uric acid homeostasis**	**Key mechanisms in inflammation modulation**	**Overall role in hyperuricemia/gout**	**Reference**
Acetate	Energy substrate for ATP, supports active excretion; May inhibit XOD indirectly; Binds/inhibits URAT1/GLUT9.	Context-dependent: may promote or inhibit NLRP3; Promotes resolution of neutrophilic inflammation.	Dual regulator; supports excretion, modulates inflammation.	Hamer et al., 2009; Vieira et al., 2015; Xu D. et al., 2019
Propionate	Inhibits URAT1, GLUT9, and SMCTs; Indirectly may affect XOD.	Inhibits NF-κB and NLRP3; Reduces ROS and pro-inflammatory cytokines.	Beneficial regulator; lowers Uric acid; anti-inflammatory.	Li Y. et al., 2022; Lu et al., 2013; Yi et al., 2022
Butyrate	Potently upregulates ABCG2; Inhibits URAT1/GLUT9; Inhibits XOD directly and via GSH.	Anti-inflammatory: HDAC inhibitor; suppresses NF-κB/NLRP3/pyroptosis; Reduces MSU-induced cytokines; Strengthens gut barrier	Central protective mediator; multi-target action.	Li et al., 2023; Kelly et al., 2015; Cleophas et al., 2016
Valerate/ Isovalerate	Elevated with beneficial interventions; Contributes to SCFA pool and energy metabolism.	Associated with anti-inflammatory and antioxidant effects; Modulates immune function.	Supportive role; contributes to overall benefits.	Deng et al., 2024; Zhou et al., 2023

ABCG2, ATP-binding cassette subfamily G member 2; GSH, glutathione; HDAC, histone deacetylase; MSU, monosodium urate; NF-κB, nuclear factor kappa-light-chain-enhancer of activated B cells; NLRP3, NOD-like receptor family pyrin domain-containing 3; SMCTs, sodium-coupled monocarboxylate transporters; XOD, xanthine oxidase.

### 4.3 Xanthine oxidase activity and intestinal barrier modulation

SCFAs exhibit the potential to directly and indirectly inhibit xanthine oxidase (XOD) activity, thereby reducing the endogenous synthesis of uric acid. Previous pharmacological studies have revealed that sodium acetate and sodium butyrate can suppress XOD activity *in vivo*, leading to a consequent decrease in serum uric acid levels in experimental models (Michael et al., 2020; Dangana et al., 2020). Butyrate has also been shown to increase hepatic concentrations of glutathione (GSH). Since xanthine dehydrogenase (XDH) can be proteolytically converted to the oxidase form, XOD, GSH inhibits this conversion, thereby indirectly reducing active XOD levels (Hamer et al., 2009). Moreover, One study proposed that serum uric acid levels were negatively correlated with serum and hepatic XOD activity, suggesting that lactic acid bacteria (LAB) reduce uric acid production by inhibiting XOD activity through SCFAs rather than degrading purines (Ni et al., 2021).

Hyperuricemia is frequently associated with intestinal barrier dysfunction and increased intestinal permeability. This promotes the translocation of bacteria and their products, particularly LPS, into the systemic circulation (Yang et al., 2024). In hyperuricemia models, this process activates the innate immune system, resulting in elevated serum levels of LPS and pro-inflammatory cytokines like tumor necrosis factor-alpha (TNF-α) (Xu D. et al., 2019). LPS itself can induce the production of other inflammatory cytokines, including interferon-gamma (IFN-γ) and interleukin-1 beta (IL-1β) (Garde et al., 1995). Critically, both LPS and these inflammatory cytokines have been demonstrated to upregulate the expression and activity of hepatic XOD, creating a vicious cycle that amplifies uric acid production (Dupont et al., 1992; Wang et al., 2019).

SCFAs counter this process by strengthening the intestinal barrier. They stabilize hypoxia-inducible factor (HIF) in intestinal epithelial cells, a transcription factor that enhances mucosal barrier function by optimizing oxygen consumption (Kelly et al., 2015). Moreover, treatment with SCFAs, especially butyrate, upregulates the expression of key tight junction proteins, such as zonula occludens-1 (ZO-1) and Occludin (Li et al., 2023). Butyrate can also promote the transcription of the tight junction protein Claudin-1 by enhancing the interaction between the transcription factor SP1 and its promoter (Wang et al., 2012). Moreover, SCFA-mediated activation of AMPK accelerates the assembly of tight junction proteins, further fortifying the intestinal barrier (Peng et al., 2009). By improving intestinal barrier integrity, SCFAs reduce the translocation of LPS to the portal circulation and liver, thereby indirectly decreasing the LPS-driven upregulation and activation of XOD, ultimately leading to reduced uric acid production.

SCFAs ameliorate hyperuricemia by suppressing XOD activity via multiple mechanisms. This highlights the potential of SCFAs as a therapeutic strategy, which operates in part by modulating the intestinal barrier and immune responses, thereby indirectly regulating uric acid metabolism.

### 4.4 SCFAs in the modulation of hyperuricemia-associated inflammation

Although only a fraction of individuals with hyperuricemia progress to symptomatic gout, it remains one of the most common causes of inflammatory arthritis worldwide (Kuo et al., 2015). The onset of gout is intrinsically linked to the activation of the innate immune system and potent inflammatory responses, which can affect not only joints but also multiple organs. High circulating levels of uric acid can induce intestinal immune dysregulation, leading to increased expression of Toll-like receptors (TLR2/4/5) and pro-inflammatory cytokines (IL-1β, TNF-α) within the intestinal mucosa (Lv et al., 2020). Concurrently, elevated levels of inflammatory mediators, including TNF-α, IL-18, IL-6, and IL-1β, are observed in the kidneys and serum of hyperuricemic mice (Liang et al., 2019). As key immunomodulatory metabolites derived from the gut microbiota, SCFAs are pivotal regulators of immunity and inflammation in various diseases and significantly shape the inflammatory landscape of hyperuricemia (Liu X. F. et al., 2023).

The NLRP3 inflammasome is a central mediator of inflammation in acute gout, and SCFAs can modulate its activation through several mechanisms. Studies have shown that sodium acetate, sodium butyrate, and sodium propionate can significantly inhibit the activation of the nuclear factor kappa-light-chain-enhancer of activated B cells (NF-κB) signaling pathway, reduce the production of reactive oxygen species (ROS), and block the NLRP3/Caspase-1 axis, thereby exerting broad anti-inflammatory effects (Yi et al., 2022). Butyrate, in particular, exerts potent anti-inflammatory actions by inhibiting histone deacetylase (HDAC) activity, which subsequently suppresses NF-κB signaling and the expression of inflammatory factors such as IL-1β, IL-2, IL-6, and TNF-α (Vinolo et al., 2011). In the context of gout, butyrate can reduce monosodium urate (MSU) crystal-induced inflammation in a concentration-dependent manner, decreasing the production of IL-1β, IL-6, and IL-8 in human peripheral blood mononuclear cells (PBMCs) (Cleophas et al., 2016). Butyrate also inhibits NLRP3-mediated pyroptosis by downregulating the NF-κB/p65 pathway, an effect that provides anti-fibrotic protection and helps preserve renal function (Tian et al., 2023). The function of acetate in inflammation appears to be context-dependent. Some studies indicate that acetate can amplify the inflammatory response to MSU crystals by increasing ROS production in immune cells through its binding to G-protein coupled receptor 43 (GPR43), thereby promoting NLRP3 inflammasome activation (Vieira et al., 2015). Conversely, other studies indicate that acetate can inhibit NLRP3 inflammasome activation by reducing intracellular calcium mobilization and, under specific conditions, promoting the polyubiquitination and autophagic degradation of the NLRP3 complex (Xu M. et al., 2019). Furthermore, SCFAs can suppress LPS-induced inflammatory responses by restoring intestinal barrier function, as previously discussed, thereby reducing the systemic load of this potent inflammagen. LPS activates the NF-κB pathway primarily through TLR4, promoting the production of pro-inflammatory cytokines (Akira et al., 2001). Butyrate has been shown to inhibit LPS-induced NF-κB activation and the subsequent transcription of pro-inflammatory genes (Segain et al., 2000). SCFAs also suppress LPS-activated NLRP3 inflammasomes by mitigating ROS production (Feng et al., 2018) and inhibit the LPS-induced elevation of serum IL-1β, IL-6, and TNF-α (Xu M. et al., 2019).

In addition to inhibiting the NF-κB/NLRP3 inflammasome axis, SCFAs exert pleiotropic effects on several other inflammatory pathways that contribute to gout pathophysiology. SCFAs can also impact innate immune cell receptor signaling. SCFAs such as acetate, propionate, and butyrate bind to G protein-coupled receptors, including FFAR2 and FFAR3, expressed on neutrophils and macrophages. Activation of these receptors modulates downstream signaling pathways and alters the production of pro-inflammatory cytokines (He et al., 2020). Complement-driven inflammation also contributes to gout pathogenesis, as MSU crystals activate the complement cascade to generate C3a and C5a, which promote leukocyte recruitment and pro-inflammatory cytokine production via C5a receptor signaling. Although direct evidence that SCFAs such as butyrate downregulate complement components or their receptors is currently limited, emerging data suggest interactions between SCFA levels and the C5a-C5aR inflammatory pathway in other inflammatory models, where modulation of this axis by SCFA supplementation is associated with attenuated inflammation, resembling the effects of C5a pathway inhibition (Szrejder and Piwkowska, 2025). Besides, SCFAs regulate immune cell recruitment and function. In neutrophils, SCFAs modulate expression of adhesion molecules and chemokine receptors, which can alter cell migration and activation states in inflamed tissues (Liu X. F. et al., 2023).

Collectively, current evidence indicates that hyperuricemia-associated inflammation is driven by a complex network involving intestinal immune dysregulation, systemic cytokine release, and activation of innate immune signaling pathways such as NF-κB and the NLRP3 inflammasome. As key microbial metabolites, SCFAs exert broad immunomodulatory effects by regulating inflammasome activation, oxidative stress, epigenetic modification, and receptor-mediated signaling, thereby shaping both local and systemic inflammatory responses. These findings highlight SCFAs as critical modulators within the gut-immune-metabolic axis and as promising targets for therapeutic intervention in gout-related inflammatory disorders.

## 5 The role of probiotics and prebiotics in hyperuricemia via SCFAs

**Figure 2:**
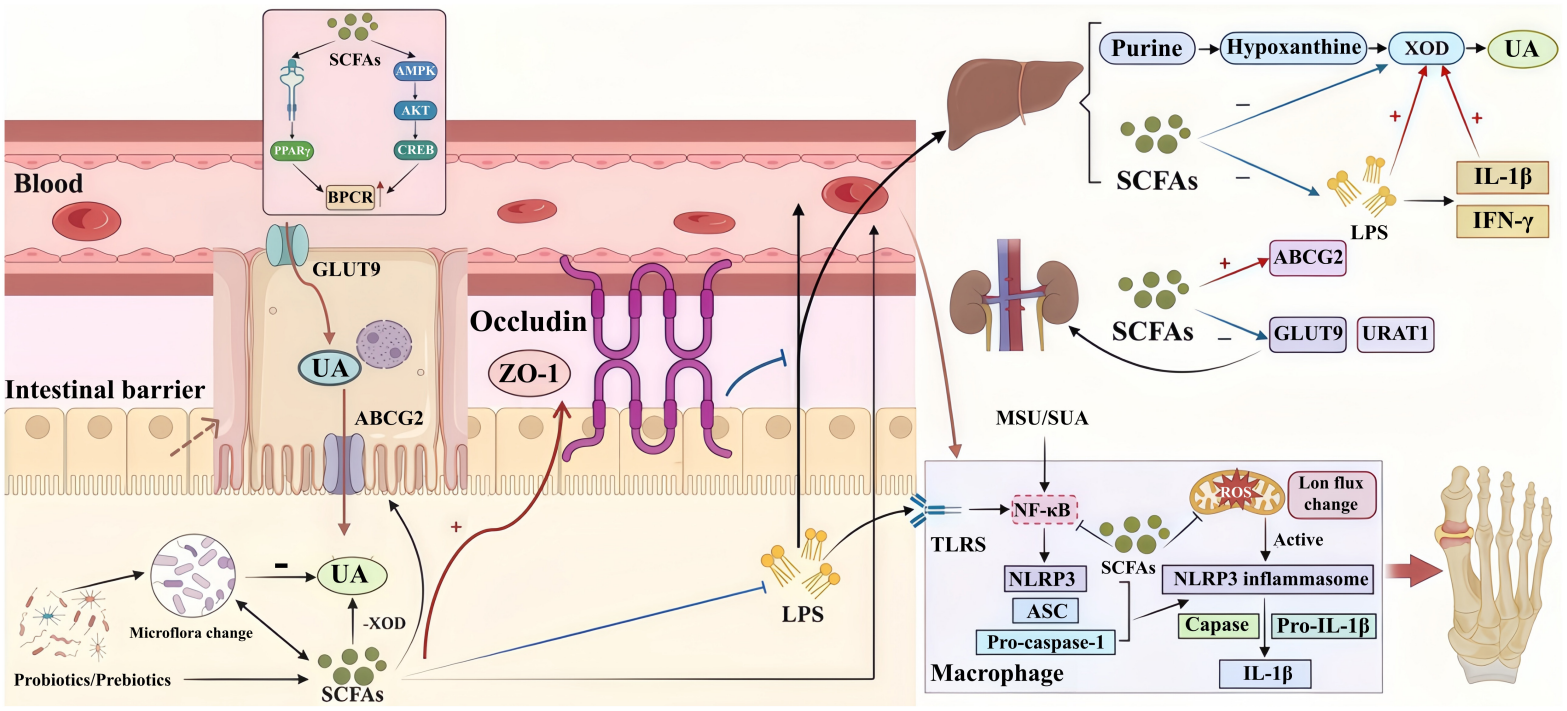
Mechanisms of short-chain fatty acids in hyperuricemia and gout. Certain prebiotics and probiotics can enhance SCFAs production through modulation of gut microbiota composition. SCFAs exert beneficial effects on hyperuricemia through multiple mechanisms, including: (1) regulation of uric acid transporter expression; (2) inhibition of urate production; (3) attenuation of inflammatory responses; and (4) restoration of intestinal barrier integrity. These collective actions contribute to the amelioration of hyperuricemia.

Conventional pharmacological treatments for hyperuricemia and gout often carry risks of hepatic and renal burden, along with adverse side effects (Sun et al., 2024). Recently, prebiotics and probiotics have gained attention as safer alternatives. Their benefit may lie in boosting SCFAs production, which helps lower serum uric acid by restoring gut microbiota balance, inhibiting uric acid synthesis, and promoting its excretion (Wang et al., 2022; Zhao et al., 2022). The application of prebiotics and probiotics thus represents a strategic approach to reestablish purine metabolic balance, with SCFAs induction constituting a central component of their mode of action, as summarized in Figure 2.

### 5.1 SCFA-mediated restoration of gut microbial ecology

The fundamental mechanism underlying all prebiotic and probiotic interventions is their capacity to restore a balanced gut microbiota composition favoring SCFAs production. Prebiotics such as *Imperata cylindrica* polysaccharide (ICPC-a) enrich SCFA-producing genera, including *Ruminococcus, Lachnospiraceae, Roseburia*, and *Bifidobacterium*, leading to increased production of acetate, butyrate, and propionate (Yu et al., 2024). Inulin supplementation increases the abundance of SCFA-producing bacteria, including *Akkermansia, Parasutterella, Ruminococcus*, and *Bifidobacterium*, resulting in elevated levels of acetate, propionate, and butyrate (Guo et al., 2021). Coffee leaf tea extracts enrich SCFA-producing taxa, including *Phascolarctobacterium, Alloprevotella*, and *Butyricicoccus*, with corresponding increases in acetate, propionate, butyrate, and valerate (Zhou et al., 2023). Probiotics, including *Lacticaseibacillus paracasei* CPU202306, increase the abundance of SCFA-producing bacteria such as *Parasutterella, Lactobacillus, Ruminococcus, Oscillospira*, and *Alistipes*, leading to elevated levels of acetic, propionic, and butyric acids (Zhou et al., 2025). *Weizmannia coagulans* BC99 enriches SCFA-producing bacteria, including *Lactobacillus* and *Akkermansia*, resulting in increased levels of acetic, propionic, and butyric acids (Wu et al., 2025).

This SCFA-mediated ecological shift creates a self-reinforcing cycle: increased SCFA production enhances intestinal barrier function, reduces inflammation, and modulates urate transporters, collectively improving the gut environment for beneficial SCFA-producing microbes. This positive feedback loop, centered on SCFA production and action, helps maintain long-term stability in uric acid homeostasis.

### 5.2 SCFA-mediated regulation of urate transporters

Prebiotics and probiotics exert significant effects on urate transport systems through their produced SCFAs (butyrate, acetate, propionate). ICPC-a elevates levels of acetate, propionate, and butyrate, and this increase in SCFAs correlates with significant upregulation of intestinal ABCG2 expression (Yu et al., 2024). Similarly, inulin supplementation increases the abundance of SCFA-producing bacteria such as *Akkermansia* and *Bifidobacterium*, resulting in elevated fecal concentrations of acetate, propionate, and butyrate, which in turn drive ABCG2 upregulation (Guo et al., 2021). Whey protein peptides PEW and LLW significantly increase the production of propionate and butyrate, and these SCFAs mediate the upregulation of ABCG2, as well as OAT1 and OAT3 (Qi et al., 2023). Probiotic strains, including *Akkermansia muciniphila* (Zhang et al., 2022), *Lactobacillus johnsonii* (Han et al., 2025), and *Lacticaseibacillus paracasei* CPU202306 (Zhou et al., 2025), all enhance intestinal SCFA levels, with butyrate subsequently activating signaling pathways such as Wnt/β-catenin and PPARγ to upregulate ABCG2 expression.

SCFAs produced under prebiotic and probiotic intervention directly inhibit renal urate reabsorption transporters through molecular interactions. Anserine supplementation elevates levels of propionate, butyrate, and valerate, and these SCFAs have been shown to physically bind to and inhibit URAT1 function (Han et al., 2021). Similarly, *Lactiplantibacillus plantarum* X7022 significantly elevates intestinal propionate and butyrate levels, which contribute to the modulation of renal urate transporters through SCFA-mediated mechanisms, including downregulation of URAT1 and GLUT9 (Zou et al., 2024). The increased levels of propionate and butyrate from whey protein peptides PEW and LLW correlate with the downregulation of both URAT1 and GLUT9 (Qi et al., 2023). Sea cucumber hydrolysates (EH-JAP and EH-LEU) elevate concentrations of propionate and butyrate, leading to the suppression of URAT1 and GLUT9 expression (Wan et al., 2020). Coffee leaf tea extracts (TE) increase multiple SCFAs, including acetate, propionate, butyrate, and valerate, collectively mediating the upregulation of ABCG2 and OAT3 alongside the downregulation of GLUT9 (Zhou et al., 2023).

### 5.3 SCFA-dependent inhibition of uric acid production

The reduction of uric acid production represents a key mechanism mediated by SCFAs induced by prebiotics and probiotics, achieved through both direct enzymatic inhibition and indirect barrier protection pathways. XOD inhibition is observed with salinomycin and *Artemisia selengensis* Turcz leaves polysaccharides (APS) and dicaffeoylquinic acids (diCQAs) treatment, which increases SCFA production, and these elevated SCFA levels correlate with significant downregulation of XOD activity (Chen et al., 2024; Lian et al., 2024). Similarly, whey protein anti-hyperuricemic fraction F5 restores acetate, propionate, and butyrate to baseline levels, underlying the observed inhibition of XOD activity (Xie et al., 2023). Coffee leaf tea extracts elevate multiple SCFAs including acetate, propionate, butyrate, and valerate, and this increase in SCFAs is associated with reduced XOD activity (Zhou et al., 2023).

Beyond direct enzyme inhibition, SCFAs produced by prebiotic and probiotic interventions indirectly suppress XOD activity through intestinal barrier restoration mechanisms. Whey protein peptide PEW elevates SCFA levels that correlate with increased expression of both occludin and ZO-1 (Qi et al., 2024), while *Pediococcus acidilactici* GR-5 enhances production of acetate, propionate, butyrate, and valerate, mediating the upregulation of intestinal barrier proteins (Ji et al., 2025). This SCFA-mediated barrier enhancement reduces LPS translocation, thereby diminishing LPS-induced XOD upregulation and creating a protective cycle where reduced endotoxemia further decreases inflammatory stimulation of XOD expression.

### 5.4 SCFA s modulation of inflammatory pathways

For individuals progressing from hyperuricemia to gout, prebiotic and probiotic interventions offer specific anti-inflammatory benefits mediated through SCFAs, utilizing both direct immunomodulatory effects and barrier-mediated pathways. Anserine supplementation elevates propionate, butyrate, and valerate, and these SCFAs suppress the formation of the NLRP3 inflammasome complex (Han et al., 2021). Salinomycin increases SCFA production, which inhibits the NF-κB/NLRP3 signaling pathway (Chen et al., 2024). Mannuronate oligosaccharides (MOS) elevate levels of acetate, propionate, and isobutyrate, which correlate with reduced production of IL-1β, a key product of NLRP3 activation (Wei et al., 2023). Probiotics such as *Lactococcus cremoris* D2022 restore SCFA concentrations, subsequently suppressing NLRP3-mediated renal inflammation (Wang et al., 2024).

The intestinal barrier restoration mediated by SCFAs contributes significantly to inflammation modulation. By strengthening the intestinal barrier through SCFA-dependent mechanisms, including upregulation of tight junction proteins via AMPK activation and HIF stabilization (Guo et al., 2021; Ji et al., 2025), these interventions reduce LPS translocation and subsequent systemic inflammation. These combined direct and barrier-mediated anti-inflammatory properties of SCFAs, provide a comprehensive mechanistic basis for the benefits of prebiotic and probiotic interventions in gout management. A comprehensive overview of the effects of various prebiotics and probiotics on hyperuricemia via gut microbiota and SCFAs modulation is provided in Table 2.

**Table 2:** Effects of prebiotics/probiotics on hyperuricemia via gut microbiota and SCFAs modulation.

**Prebiotics/ probiotics**	**Category**	**Sources**	**Mode of action/mechanism**	**Changes in SCFA-producing microbiota**	**Changes in SCFA levels**	**Reference**
ICPC-a	Prebiotics	*Imperata cylindrica*	ABCG2↑; ameliorat the reduction in colon length	*Ruminococcus*↑, *Lachnospiraceae*↑, *Roseburia*↑, *Bifidobacterium*↑	Acetate↑, butyrate↑, propionate↑	Feng et al., 2018
Anserine	Prebiotics	Tuna red muscle	ADA↓, XOD↓, URAT1↓, NLRP3 inflammasome complex↓; inhibit the TLR4/MyD88/NF-κB signaling pathway	*Lactobacillus*↑, *Clostridium*↑, *Bacteroides*↑	Propionate↑, butyrate↑, valerate↑	Han et al., 2021
Salinomycin	Prebiotics	*Streptomyces albus*	ABCG2↑; inhibit the NF-κB/NLRP3 signaling pathway	*Alistipes*↓, *Bacteroidales*↑	SCFAs↑ (except hexanoic acid)	Chen et al., 2024
F5	Prebiotics	Whey protein	OAT1↑, XOD↓, TNF-α↓, IL-1β↓, IL-6↓, URAT1↓, GLUT9↓	*Bacteroides*↑, *Rikenellaceae*_RC9_gut_group↑, *Ruminococcaceae*_UCG-014↑	Acetate↑, propionate↑, butyrate↑	Xie et al., 2023
APS and diCQAs	Prebiotics	*Artemisia selengensis* Turcz	XOD↓	*Bacteroidetes*↑, *Firmicutes*↑, *Lactobacillus*↑	Acetate↑, butyrate↑, propionate↑, isovalerate↑	Lian et al., 2024
Inulin	Prebiotics	Root vegetables	ABCG2↑, occludin↑, ZO-1↑, XOD↓, LPS↓, TNF-α↓, IL-1β↓, IL-6↓	*Akkermansia*↑, *Parasutterella*↑, *Ruminococcus*↑, *Bifidobacterium*↑	Acetate↑, propionate↑, butyrate↑	Guo et al., 2021
TE	Prebiotics	Coffee leaf tea	ABCG2↑, OAT3↑, XOD↓, LPS↓, GLUT9↓	*Phascolarctobacterium*↑, *Alloprevotella*↑, *Butyricicoccus*↑	Acetate↑, propionate↑, butyrate↑, valerate↑	Zhou et al., 2023
KGM	Prebiotics	Konjac	XOD↓	*Lachnoclostridium*↑, *Lachnospiraceae*↑	Acetate↑, butyrate↑, valerate↑, isobutyrate↑, isovalerate↑	Deng et al., 2024
PEW and LLW	Prebiotics	Whey protein	ABCG2↑, OAT1↑, OAT3↑, GLUT9↓, URAT1↓	*Lactobacillus*↑, *Ruminococcus*↑, *Bifidobacterium*↑	Acetate↑, butyrate↑, valerate↑	Qi et al., 2023
PEW	Prebiotics	Whey protein	ABCG2↑, XOD↓, TNF-α↑, IL-1β↑, IL-6↑, GLUT9↑, occludin↑, ZO-1↑	*Muribaculaceae*↑, *Lactobacillus*↑, *Ruminococcus*↑	Acetate↑, butyrate↑, valerate↑	Qi et al., 2024
EH-JAP and EH-LEU	Prebiotics	*Apostichopus japonicus* and *A. leucoprocta*	ABCG2↑, XOD↓, OAT1↑, MRP4↑; inhibit the TLR4/MyD88/NFκB signaling pathway	*Lactobacillus*↑, *Clostridiales*↑, *Ruminococcaceae*↑	Propionate↑, butyrate↑	Wan et al., 2020
EPP	Prebiotics	*Enteromorpha prolifera*	ABCG2↑, XOD↓, URAT1↓, OAT1↑	*Alistipes*↑	Butyrate↑	Li et al., 2021
MOS	Prebiotics	Kelp and *Undaria pinnatifida*	ABCG2↑, URAT1↓, IL1β↓, GLUT9↓, IL-12↓, IL-18↓	*Muribaculum*↑, *Ruminococcus*↑, *Faecalibaculum*↑	Acetate↑, propionate↑, isobutyrate↑, isovalerate↑	Wei et al., 2023
*A. muciniphila*	Probiotics	Human gut microbiota	ABCG2↑, XOD↓, URAT1↓, tight junction proteins↑, TNF-α↓, IL-1β↓, IL-6↓, Caspase1↓, GLUT9↓	*Muribaculum*↑, *Ruminococcus*↑, *Oscillospira*↑, *Prevotellaceae*-UCG-001↑, *Alistipes*↑	Acetate↑, propionate↑, butyrate↑, valerate↑	Zhang et al., 2022
*Lactiplantibacillus plantarum* X7022	Probiotics	Fermented stinky tofu	XOD↓, URAT1↓, OAT1↑, GLUT9↓	*Bifidobacteria*↑, *Rikenellaceae*_RC9_gut_group↑, *Lactiplantibacillus*↑	Propionate↑, butyrate↑	Zou et al., 2024
*Pediococcus acidilactici* GR-5	Probiotics	Jiangshui	ABCG2↑, OAT1↑, occludin↑, ZO-1↑, XOD↓, ADA↓, URAT1↓, GLUT9↓; inhibit the NLRP3 pathway	*Lactobacillus*↑, *Seramator thermalis*↑, *Prevotella*↓	Acetate↑, propionate↑, butyrate↑, valerate↑	Ji et al., 2025
*Lactobacillus johnsonii*	Probiotics	Human gut microbiota	ABCG2↑, XOD↓	*Lactobacillus*↑	Butyrate↑	Han et al., 2025
*Lacticaseibacillus paracasei* CPU202306	Probiotics	Fermented pickles	ABCG2↑, OAT3↑, tight junction proteins↑, XOD↓, ADA↓, URAT1↓, GLUT9↓; inhibits the TLR4/MyD88/NF-κB pathway	*Parasutterella*↑, *Lactobacillus*↑, *Ruminococcus*↑, *Oscillospira*↑, *Alistipes*↑	Acetate↑, propionate↑, butyrate↑, valerate↑	Zhou et al., 2025

ICPC-a, Imperata cylindrica polysaccharide; ABCG2, ATP-binding cassette subfamily G member 2; ADA, adenosine deaminase; XOD, xanthine oxidase; URAT1, uric acid transporters1; NLRP3, NOD-like receptor family, pyrin domain containing 3; TLR4, toll-like receptor 4.; MyD88, myeloid differentiation factor 88; NF-κB, nuclear factor-κB; OAT1, organic anion transporter 1; TNF-α, tumor necrosis factor-alpha; IL-1β, interleukin-1 beta; IL-6, interleukin-6; GLUT9, glucose transporter 9; APS, Artemisia selengensis Turcz leaves polysaccharides; diCQAs, dicaffeoylquinic acids; ZO-1, zonula occludens-1; LPS, lipopolysaccharide; TE, coffee leaf tea extracts; KGM, konjac glucomannan; PEW, Pro-Glu-Trp; LLW, Leu-Leu-Trp; OAT3, organic anion transporter 3; EH-JAP, A. japonicus; EH-LEU, A. leucoprocta; MRP4, multidrug resistance protein 4; EPP, E. prolifera polysaccharides; MOS, mannuronate oligosaccharide; IL-12, interleukin-12; IL-18, interleukin-18; A. muciniphila, Akkermansia muciniphila.

## 6 Conclusions and future perspectives

The mechanistic exploration of intestinal involvement in uric acid metabolism has fundamentally reshaped the understanding of hyperuricemia, establishing the gut microbiota and its metabolites as pivotal determinants of systemic urate homeostasis. At the heart of this regulatory network lies the gut microbiota-SCFA-urate axis. This review synthesizes evidence demonstrating that interventions with prebiotics and probiotics ameliorate hyperuricemia and gout through a central, shared mechanism: the production of SCFAs. These microbially derived metabolites, in turn, function as pivotal effector molecules that orchestrate a multi-faceted therapeutic response. They act by modulating the expression and activity of urate transporters in the kidneys and intestines to facilitate excretion, suppressing xanthine oxidase activity to limit production, strengthening the intestinal barrier to reduce the systemic influx of pro-inflammatory molecules, and directly inhibiting key inflammatory pathways such as the NLRP3 inflammasome. Consequently, strategies aimed at modulating the gut microbiota to enhance SCFAs production present a viable, multi-targeted, and low-toxicity framework for the management of hyperuricemia and the prevention of its associated complications.

Future research should focus on several key directions: first, more physiologically relevant models such as intestinal organoids should be utilized to elucidate the precise molecular mechanisms by which SCFAs regulate intestinal uric acid homeostasis. Second, the differential effects of varying SCFA ratios on hyperuricemia progression should be systematically investigated to clarify their synergistic or antagonistic interactions. Third, well-designed randomized controlled trials are needed to directly validate whether SCFAs are indeed the key mediators of the beneficial effects of prebiotic/probiotic interventions in hyperuricemia. Additionally, research on intestinal urate transporters still lags behind that of renal transporters and requires more in-depth exploration. Meanwhile, the role of SCFAs in modulating the NLRP3 inflammasome pathway remains controversial, and their context-dependent effects need further clarification. Finally, the mechanisms of action of prebiotics and probiotics are complex and multifaceted, and the specific contribution of SCFAs within these networks requires more precise delineation. These investigations will provide evidence-based support for targeted dietary interventions and personalized treatment approaches.
